# Natural history of non-bullous impetigo: a systematic review of time to resolution or improvement without antibiotic treatment

**DOI:** 10.3399/bjgp20X714149

**Published:** 2021-02-09

**Authors:** Tammy C Hoffmann, Ruwani Peiris, Paul Glasziou, Gina Cleo, Chris Del Mar

**Affiliations:** Institute for Evidence-Based Healthcare, Faculty of Health Sciences and Medicine, Bond University, Gold Coast, Queensland.; Institute for Evidence-Based Healthcare, Faculty of Health Sciences and Medicine, Bond University, Gold Coast, Queensland.; Institute for Evidence-Based Healthcare, Faculty of Health Sciences and Medicine, Bond University, Gold Coast, Queensland.; Institute for Evidence-Based Healthcare, Faculty of Health Sciences and Medicine, Bond University, Gold Coast, Queensland.; Institute for Evidence-Based Healthcare, Faculty of Health Sciences and Medicine, Bond University, Gold Coast, Queensland.

**Keywords:** general practice, impetigo, natural history, primary care

## Abstract

**Background:**

Non-bullous impetigo is typically treated with antibiotics. However, the duration of symptoms without their use has not been established, which hampers informed decision making about antibiotic use.

**Aim:**

To determine the natural history of non-bullous impetigo.

**Design and setting:**

Systematic review.

**Method:**

The authors searched PubMed up to January 2020, as well as reference lists of articles identified in the search. Eligible studies involved participants with impetigo in either the placebo group of randomised trials, or in single-group prognostic studies that did not use antibiotics and measured time to resolution or improvement. A modified version of a risk of bias assessment for prognostic studies was used. Outcomes were percentage of participants who had either symptom resolution, symptom improvement, or failed to improve at any timepoint. Adverse event data were also extracted.

**Results:**

Seven randomised trials (557 placebo group participants) were identified. At about 7 days, the percentage of participants classified as resolved ranged from 13% to 74% across the studies, whereas the percentage classified as ‘failure to improve’ ranged from 16% to 41%. The rate of adverse effects was low. Incomplete reporting of some details limited assessment of risk of bias.

**Conclusion:**

Although some uncertainty around the natural history of non-bullous impetigo remains, symptoms resolve in some patients by about 7 days without using antibiotics, with about one-quarter of patients not improving. Immediate antibiotic use may not be mandatory, and discussions with patients should include the expected course of untreated impetigo and careful consideration of the benefits and harms of antibiotic use.

## INTRODUCTION

Antibiotics may make little clinical difference in many of the self-limiting infections with which patients present to general practice. Non-bullous impetigo (also known as school sores) is a common skin infection caused by bacteria such as *Staphylococcus aureus* and *Streptococcus pyogenes*, and is often treated with antibiotics, given either topically or orally.^1^ Common antibiotics used include fusidic acid, mupirocin, flucloxacillin, and clarithromycin. Rates of bacterial resistance to the antibiotics used to treat impetigo have been increasing worldwide.^2^^,^^3^

The rationale for antibiotic use is that they are thought to reduce the risk of contagious spread within the patient and to others, speed up resolution of the lesions, and reduce the risk of complications such as rheumatic fever in vulnerable populations. However, these antibiotics may cause side effects, as well as contribute to the development of antibiotic resistance,^4^ and use should be limited where possible. The natural history of impetigo when antibiotics are not used is unclear.

Knowledge of the natural course of impetigo, including the likely duration of symptoms, can assist in informed decision making about management of the condition. This information can assist clinicians to engage with patients in shared decision making, and help them to have a better understanding of what may happen if antibiotics are not used compared with what happens if they are.^3^^,^^5^ This study aimed to review all published information available from placebo groups of randomised trials or from cohort studies about the time to resolution of non-bullous impetigo symptoms without antibiotic treatment.

## METHOD

### Eligibility criteria

The authors aimed to identify studies that met the following criteria:

- included children or adults who had non-bullous impetigo;
- reported outcome data on the clinical improvement and/or resolution of non-bullous impetigo over time, and
- included a group that received no therapeutic treatment (that is, placebo or ‘no treatment’) — this could be either a comparison group in a randomised trial or a single-group prognosis study, such as a cohort study.

### Search methods

A librarian experienced in systematic reviews conducted the search in PubMed up to 15 November 2018 to identify systematic reviews (as a method of identifying potentially eligible randomised trials) and prognosis studies (see Supplementary Box S1). A forward–backward citation analysis was also performed. An updated search was conducted on 28 January 2020, but found no further eligible studies.

**Table:** How this fits in

Non-bullous impetigo is a very common reason for general practice consultation and antibiotic prescription. Informed decision making should consider the benefit–harm trade-off of antibiotic use and the natural course of the illness. Non-bullous impetigo resolves spontaneously in some patients by about 7 days. Antibiotics may not always be necessary for all patients immediately.

### Screening and eligibility assessment

One researcher screened the search results by title and abstract, and then checked the full text of potentially eligible articles; discussions with a second researcher about eligibility took place as needed. Three non-English articles that were potentially eligible were translated for screening purposes using Google Translate (two were excluded at abstract level, and one after full-text check).

### Risk of bias assessment and data extraction

As the authors were focused on using prognosis outcome data, they used a modified version of a risk of bias assessment framework proposed by Altman *et al* to assess the included studies.^6^ The study and methodological quality characteristics that were extracted were: country, sample size, age, inclusion criteria, exclusion criteria, definition of ‘clinical cure’ and ‘failure to improve’, randomisation method, placebo, concurrent treatment, symptom duration before study inclusion, and duration of follow-up.

To obtain data regarding the proportion of symptom-free patients, the authors extracted data from the studies, either directly from the published text and tables or, where needed, by using extraction software (WebPlotDigitizer, https://automeris.io/WebPlotDigitizer) to retrieve values from the figures. In trials in which participants’ symptoms worsened and they left the placebo group to commence antibiotics, the authors calculated the percentage outcome data by using the total number of participants that were initially randomised to the placebo group as the denominator, whenever it was possible to do so.

### Outcomes

Data were extracted for the following outcomes from participants in the placebo group, along with the study authors’ definition of each:

- percentage of participants with resolved symptoms (‘clinical cure’) at any timepoint;
- percentage of participants clinically improved at any timepoint; and
- percentage of participants whose symptoms failed to improve (requiring antibiotics) at any timepoint.

Data were also extracted regarding the rate of crossover from placebo to antibiotic groups, reasons for the crossover, and adverse events within both treatment and placebo group participants. In some studies, percentage values were given for a time range (for example, x% symptom free at 7–9 days). In these cases, the median timepoint was used to present values graphically (for example, x% symptom free at 8 days).

### Data analysis

Scatter plots of outcome data versus time were used to enable visualisation of the rate of symptom resolution. The diameter of the data points on the scatter plots represents each study’s sample size.

## RESULTS

**Figure 1.**
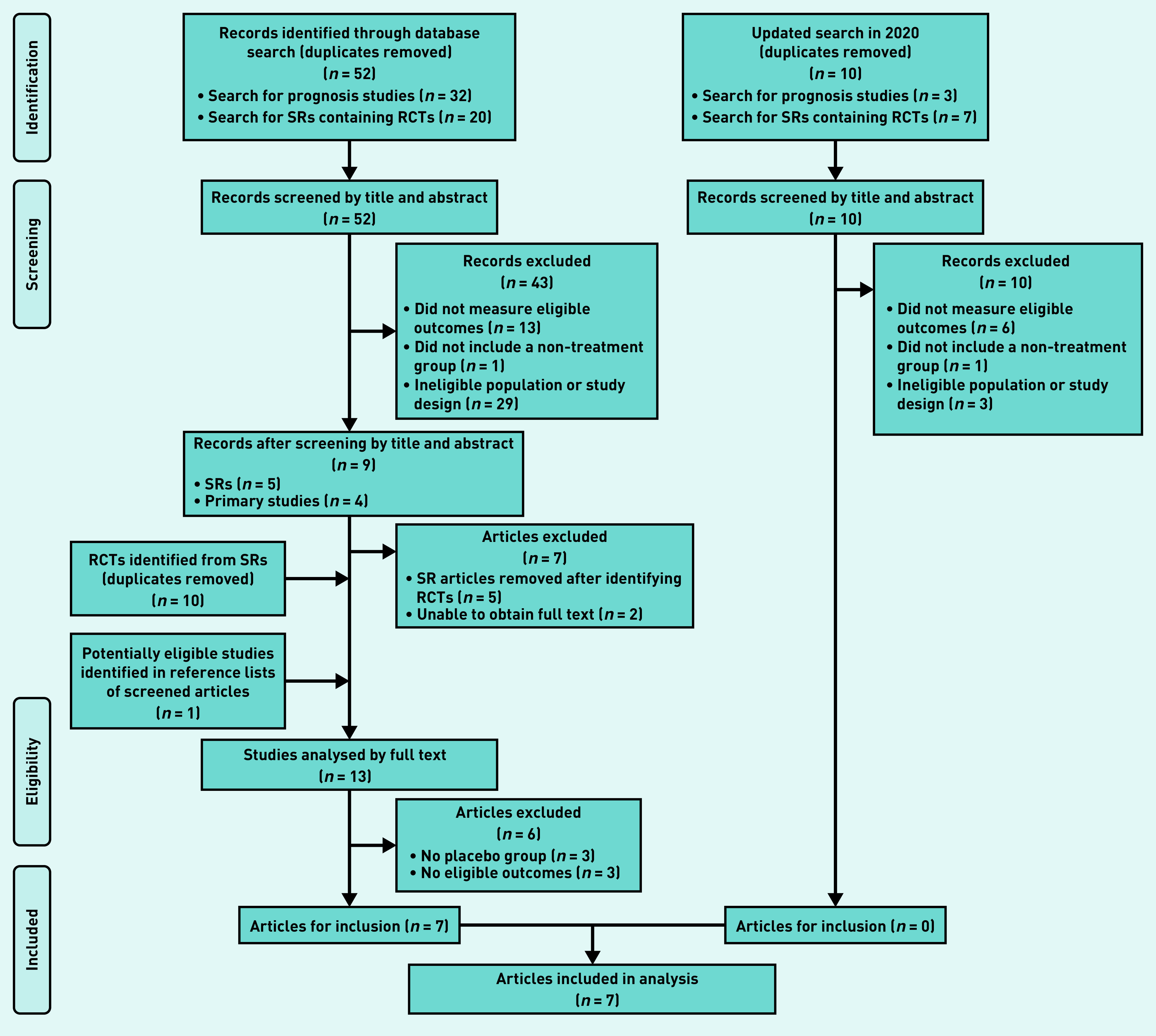
***Flow of articles through the review.*** ***RCT = randomised controlled trial. SR = systematic review.***

Figure 1 shows the flow of articles. The authors found 52 studies from the database searches and one additional study from citation searching of identified relevant studies. After full-text checking, seven studies were included.^7^^–^^13^ The updated search conducted in January 2020 identified 10 articles for screening, but no new eligible studies.

### Characteristics of included studies

All studies were randomised controlled trials, with the size of the placebo groups ranging from 12 to 206 participants (see Supplementary Table S1). The age across studies ranged from infancy to 80 years, with three trials including only children. There were no common exclusion criteria across all studies, although most reported recent antibiotic use as a reason for exclusion. Two studies excluded participants with eczema. The criteria for assessing clinical cure varied; some studies required complete disappearance of skin changes, whereas others required only that lesions be dry. Two studies used a Skin Infection Rating Scale (SIRS) and required healed lesions, but allowed for limited ongoing skin changes.

The placebos used were topical creams (four studies), ointments (two studies), and an oral suspension (one study). In three studies, placebo group participants were also instructed to use a concomitant treatment (such as, povidone-iodine shampoo, hexachlorophene soap, and Castile bath soap), and one study had ‘allowed treatments’ that participants could use, although these were not specified.

### Risk of bias of included studies

The risk of bias assessment is shown in Table 1. Most studies reported on at least two of the four criteria. Randomisation and blinding were variably and often incompletely reported, making the risk of potential selection and ascertainment bias difficult to determine.

**Table 1.** Risk of bias assessment for the included studies

	**Ruby, 1973^7^**	**Zaynoun, 1974^8^**	**Eells, 1986^9^**	**Koning, 2002^10^**	**Koning, 2008^11^**	**Gropper, 2014^12^**	**Rosen, 2018^13^**
**Defined sample: description of source of patients and inclusion and exclusion criteria**	Hospital outpatient clinic. Describes some exclusion criteria, but not inclusion criteria	Hospital outpatient clinic. Does not describe inclusion or exclusion criteria	No mention of where trial was conducted. Describes inclusion and exclusion criteria	General practices. Describes inclusion and exclusion criteria	‘General practices and dermatology departments’. Describes inclusion and exclusion criteria	Unclear patient source: ‘27 centres in five countries’. Mentions ‘clinic visits’. Describes inclusion and exclusion criteria	Unclear patient source: multicountry and multicentre trial at various sites (clinic, hospital). Describes inclusion and exclusion criteria
**Representative sample: participants were selected as consecutive cases**	NR	NR	NR	NR	NR	NR	NR
**Follow-up rate: outcome data available for ≤80% of participants at one follow-up point**	NR	Yes	Yes	Yes	79% (*n* = 58/73) on day 14, per-protocol	Yes	Yes (but unequal loss between groups; more loss in placebo group)
**Prognosis: raw data, percentages, survival rates, or continuous outcomes reported**	Yes	Yes	Yes	Yes	Yes	Yes	Yes

NR = not reported.

### Clinical resolution or clinical improvement over time

**Figure 2.**
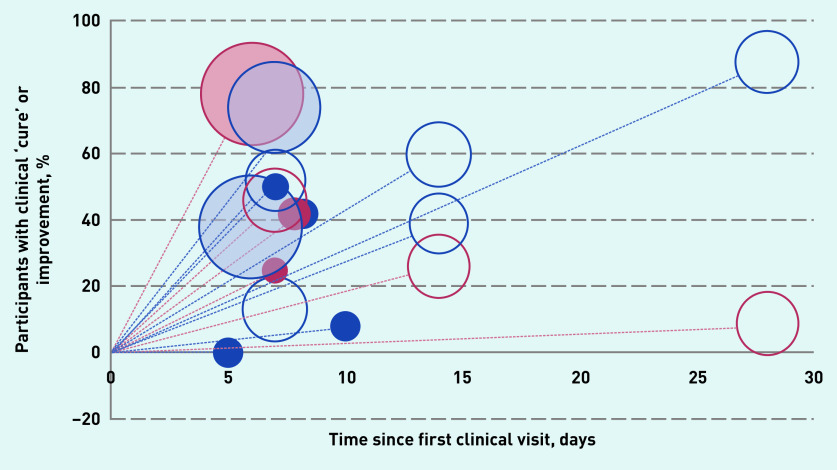
***Change over time in the percentage of placebo participants with clinical cure (blue circles) or clinical improvement (orange circles).****^a^* *^a^****Size of circles corresponds to sample size of each study. Varied definitions of cure and improvement as used are represented by: filled circle = complete skin resolution; empty circle = clearance of infection but skin changes can be present; partially filled circle = intermediate clearance. Dotted lines highlight the change from an initial 0% cure or improvement rate at day 0 (see Supplementary Table S2).***

Eleven values for clinical resolution and six values for clinical improvement were extracted (see Supplementary Table S2 and Figure 2). Most studies made an assessment around day seven (counting from the first clinical visit). At around day seven, the percentage of participants with clinical cure or improvement ranged from 13% to 74%. As this wide range could be affected by the various definitions for ‘clinical cure’, data points in Figure 2 are colour coded to represent complete clearance (lesions resolved and no skin changes seen), infection clearance (lesions healed but skin changes may still be present), and intermediate clearance (difficult to tell from study definition if skin changes are present) *.* This colour coding does not reveal a pattern of symptom resolution related to definition of ‘cure’/improvement.

### Failure to improve and adverse effects over time

**Figure 3.**
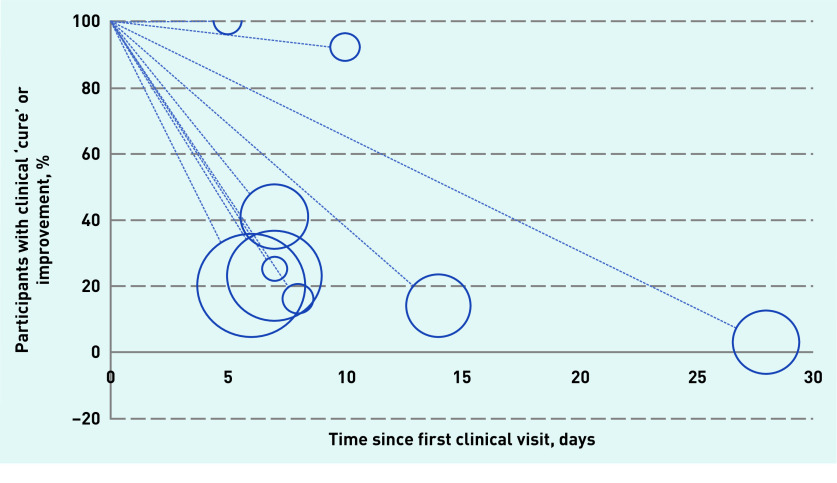
***Change over time in the percentage of placebo participants who ‘failed to improve’.****^a^* *^a^****Dotted lines highlight the change from an initial 100% ‘failure rate’ at day 0 (see Supplementary Table S2).***

Nine values for clinical failure to improve were extracted (see Supplementary Table S2 and Figure 3). Lines drawn from the 100% failure to improve mark at 0 days (the day of first clinical visit) to each data point help to visualise the resolution of symptoms among participants towards zero symptoms. Five values were measured 6–8 days after the first clinical visit, when the lowest percentage of participants with failure to improve was 16% and the highest was 41%. The rate of crossover or commencement of antibiotics at 1 week was reported in four studies. No major adverse effects for placebo participants were reported in any study.

## DISCUSSION

### Summary

The paucity of data for the natural history of impetigo has been noted previously.^3^ In this review, data from the placebo groups of seven randomised trials were used. No prognostic cohort studies were identified. For all studies, the focus of the outcome measurement timing was at or about 7 days. The percentage of participants classified as cured by about 7 days ranged from 0% to 74% across the studies, with about half of the studies providing an estimate that approximately half of the participants were cured. Conversely, the percentage of participants who were classified as failure to improve by about 7 days ranged from 16% to 41%, with three studies providing an estimate of about one-quarter of the participants.

### Strengths and limitations

This review examined the natural history data for this condition that were available from placebo-controlled trials, a novel approach to providing prognostic information that is important for clinical decision making. It gives some evidence of what to expect when neither topical nor oral antibiotics are used. However, topical disinfectants were allowed in both intervention and placebo groups in four of the studies, which may have influenced the natural course of impetigo in cases where no intervention was used.

A possible limitation is that the authors searched for systematic reviews as a method of identifying relevant randomised trials. This pragmatic approach was used because systematic reviews of trials of interventions for impetigo exist that would have already identified potentially eligible trials. Heterogeneity, such as in the definition of cured/healed lesions and timing of outcome measurement, precluded a meta-analysis being conducted. Other limitations in the available data include that the inclusion and exclusion criteria varied among the studies (for example, two studies included some bullous forms and not all explicitly excluded eczema, which can be difficult to distinguish from impetigo); duration of symptoms before inclusion in the study was unclear for all but two studies; and the use of concomitant treatments was allowed in four studies. Incomplete reporting of methods such as case selection and method of blinding for many studies limited assessment of prognosis-related risk of bias.

### Comparison with existing literature

The opinion that impetigo lasts about 2–3 weeks without treatment is provided in the introduction of a Cochrane systematic review of interventions for impetigo and in clinical guides,^3^^,^^14^ but this does not appear to be informed by research. The Cochrane review found that the cure rate in placebo groups at 7 days ranged from 0% to 42%.^3^ The current review includes two more recent randomised trials, hence the higher cure rates in the range. Interpretation of ‘clinical cure’ values is made difficult by the differences in the definition across the studies. Although some required a complete return to normal skin, others required only that the lesions be ‘inactive’, allowing for ongoing signs of skin inflammation. However, all definitions included the stipulation that the lesions be dry, and that exudate or pus should be absent.

### Implications for research and practice

Certainty about the natural history of non-bullous impetigo would be increased by additional primary research — ideally, rigorous studies with appropriate sample sizes, follow-up beyond the first week of symptoms, use of standardised outcome measure description and timing, and measurement of recurrence and spread to others such as family members.

This review provides some data showing that the use of antibiotics is not mandatory for patients with non-bullous impetigo. Information about the expected course of untreated impetigo may help patients and parents to frame their expectations about recovery timeframes. Even if a precise mean duration of time to healing is not available from existing data, the information presented may be useful for primary care clinicians to engage in shared decision making with patients (or their parents) about whether antibiotics should be used for the management of impetigo. Given the cure rates at 1 week suggested by this review, adopting a delayed prescribing approach to antibiotics may be appropriate for many patients with impetigo. None of the included studies reported serious adverse effects in the placebo group participants, and the rate of any minor adverse effects was low. In certain populations, non-suppurative complications (for example, acute glomerulonephritis and acute rheumatic fever), which may be caused by *Streptococcus*, a common causative agent associated with impetigo, are more likely.^15^^,^^16^
